# Eosinophilic esophagitis (EoE); a disease that must not be neglected - implications of esophageal rupture and its management

**DOI:** 10.1186/s12876-020-01330-y

**Published:** 2020-06-11

**Authors:** Helen Larsson, Stephen Attwood

**Affiliations:** 1 Department of Otorhinolaryngology, NU-Hospital Group, Trollhättan, Sweden; 2 Durham University, Durham, United Kingdom

**Keywords:** Eosinophilic esophagitis, Esophageal perforation, Esophageal bolus obstruction

## Abstract

**Background:**

The prevalence of Eosinophilic esophagitis (EoE) is increasing, a severe complication of EoE is spontaneous perforation of the oesophagus. It is of great importance to be aware of this risk and handle this severe complication carefully.

**Case presentation:**

A middle-age man with EoE since 2004, had a total esophageal bolus obstruction while eating lunch at the local hospital. Drinking water, in an attempt to release it, led to a total intramural ruptur of the esophageal wall. A CT scan detected the injury and a covered esophageal stent was inserted within 2 h from the injury. Despite the immediate hospital care, he developed mediastinitis, were in need of a laparascopy and intensive care. After 8 weeks the stent was removed and the esophagus was considered healed. Biopsies from the esophagus showed an eosinophilic inflammation (65 eosinophils/HPF). Twelve weeks from the injury he was essentially back in his normal state and was discharged from the hospital. He was placed on a 6 weeks course of topical treatment with budesonide, which needed to be extended due to inadequate remission. Remission was achieved after 12 weeks of treatment.

**Conclusion:**

An effective topical steroid treatment in EoE patients is important. EoE patients are in risk of oesophageal perforation, if so, management may be conservative but mediastinal drainage is important if significant extravasation occurs and should be instituted from the start.

## Background

EoE is a chronic, local, immune-mediated esophageal disease, characterized clinically by symptoms related to esophageal dysfunction and histologically by eosinophil-predominand inflammation, with increasing incidence [1, 2]. Over time many EoE-patients develop an impaired distensibility of the esophageal wall and esophageal fibrostenosis caused by prolonged inflammation [3].

The most common symptom for adult EoE-patients is esophageal dysphagia, which not infrequently leads to total food bolus obstruction. In an attempt to release it, the patient typically tries to drink water or vomit to get the bolus to pass. If the obstruction does not spontaneously improve, the patient is in need of emergency medical care. Then the foreign body needs to be removed endoscopically. An unusual, but well known, complication caused by EoE is rupture of the esophageal wall [4]. However, there are only few reports on the coexistence of this with food bolus obstruction [5].

## Case presentation

A 52 –year-old man, medically experienced as a physician in medicine was diagnosed with EoE 2004. At the time of diagnosis he was treated with oral systemic corticosteroids (according to the practice at that time) and then later with topical treatment with corticosteroids (mometasone furoate 800 μg/ day) for 2 months period. Since then, the patient has been self-treating, using orally sprayed formula of mometasone furoate in lower doses, according to degree of dysphagia. But above all he has modified the food consistency and added large amounts of water during food intake. The patient chose not to attend medical follow up but to manage his own symptoms over the interval of 14 years.

In Nov 2018, at lunch in the hospital at which he worked, he suffered a food bolus obstruction which did not release with drinking water. He suffered a sudden and severe thoracic pain, and he consciously realised that his esophagus had perforated.”

**Fig. 1:**
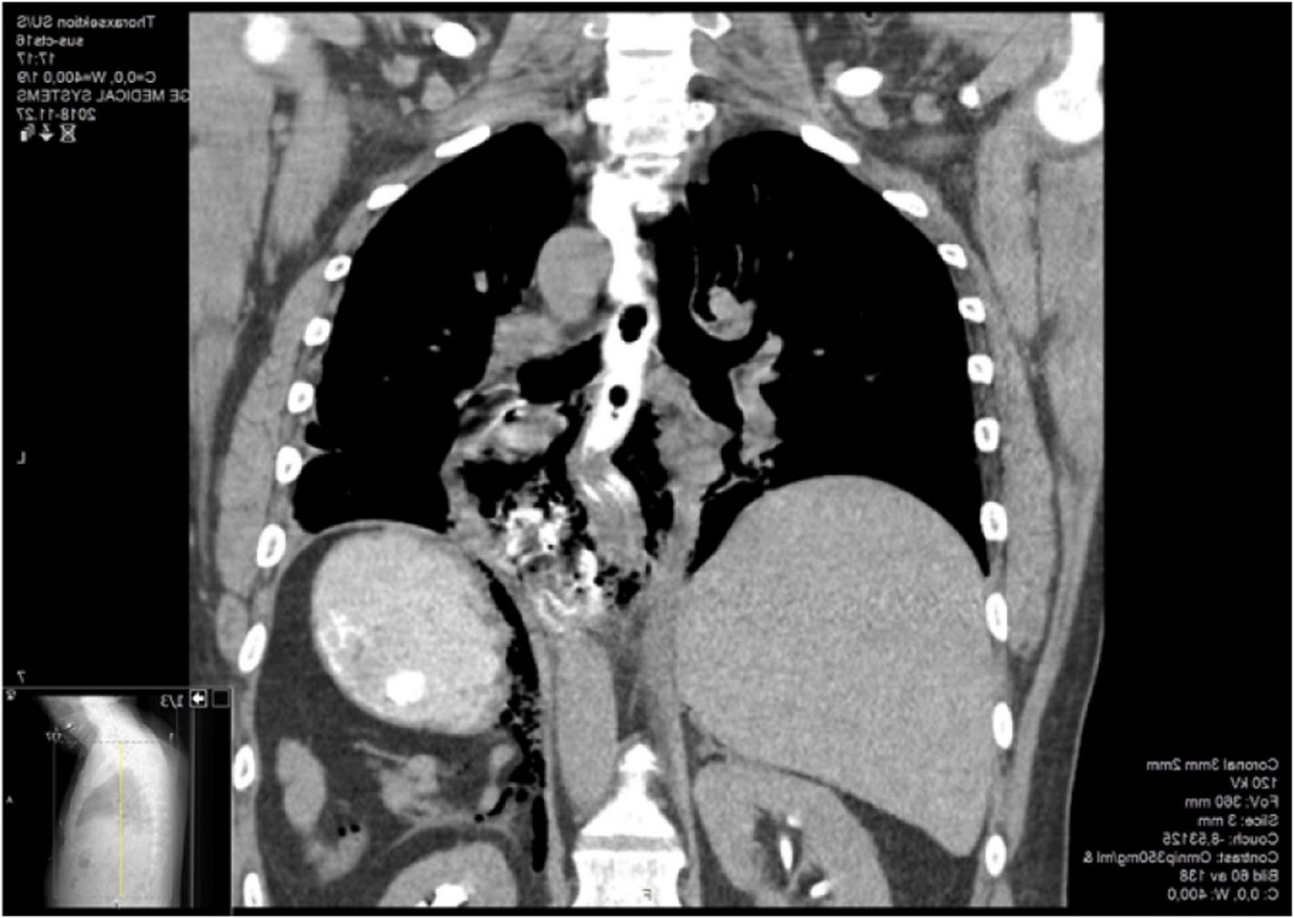
CT image, about 1 h after the injury; demonstrates air in the mediastinum and esophageal rupture

This was verified with an acute CT scan that detected air in the mediastinum and signs of esophageal rupture (Fig. 1). The patient was flown to the nearby university hospital where an endoscopy was performed and a complete intramural esophageal rupture of approximately 2 cm, and a 5 cm mucosal tear, in the middle part of the esophagus was detected. A covered stent was inserted within 2 h from the injury. The next day the patient’s condition worsened due to mediastinitis and abscess development, laparoscopy was performed with several thoracic and abdominal drainages.

**Fig. 2:**
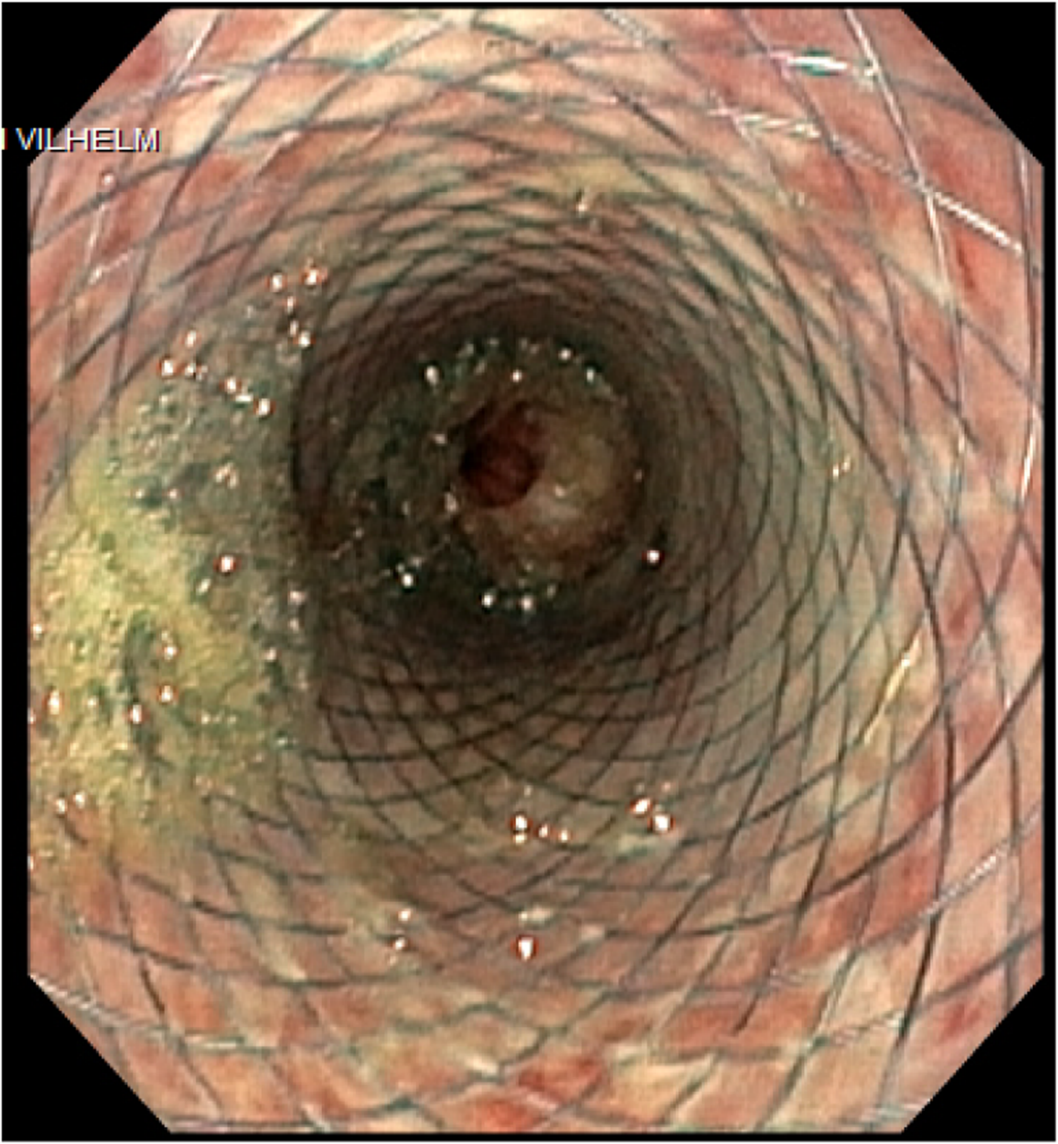
Stent in the esophagus, before removal after 8 weeks of treatment

After 8 weeks of intravenous broad-spectrum antibiotics, initially antifungal medication, and a feeding jejunostomy the esophageal stent was removed and the esophageal wall assessed endoscopically as being healed (Fig. 2).

**Fig. 3:**
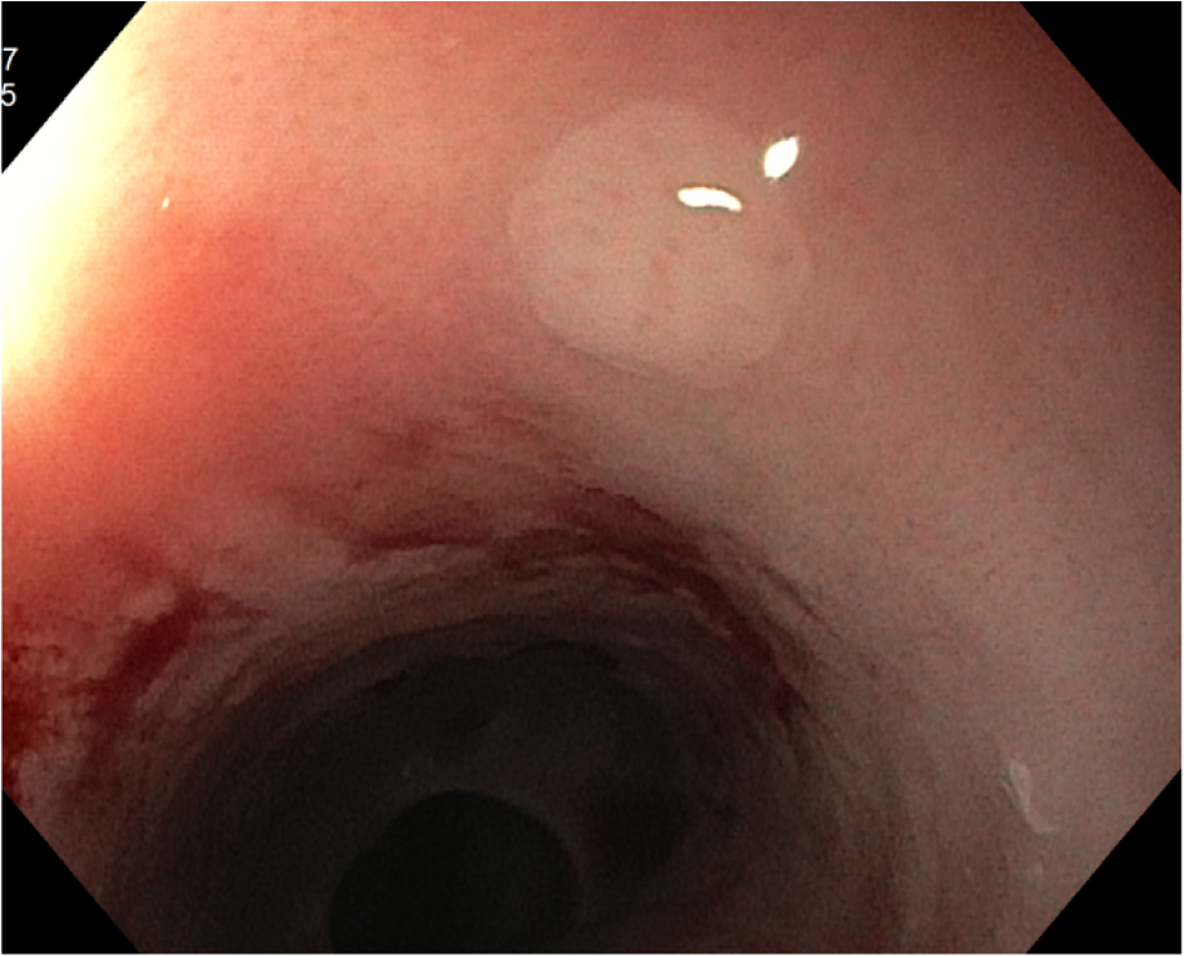
White plaques in proximal esophagus, photo after biopsy

Postoperatively he suffered from attacks of kidney stones (a pain which, according to the patients was negligible compared to the pain he felt in the acute phase of the esophageal rupture), severe nausea and a minor stroke, altogether leading to a prolonged hospital stay. The nausea was considered to be linked to a gastroparesis, suspected to be caused by a damage to the vagus nerve, and was treated with erythromycin injections, as well as botulinum toxin injections in the pylorus, in the same session biopsies were taken from the esophageal mucosa showing a remaining eosinophilic inflammation (peak value 65 eosinophils/HPF; 284 eosinophils/mm^2^) (Fig. 3).

The patient was discharged from the hospital after 85 days, essentially back in his normal state, but with 25% less body weight. The patient experienced only mild esophageal dysphagia, probably due to the previous stent treatment. The esophageal inflammation was treated with a 6 weeks course with Budesonide orodispersble tablet (BOT) 1 mg two times a daily, due to inadequate histological remission (45eosinophils/HPF) the treatment was extended. After 12 weeks of treatment remission was achieved (5 eosinophils/HPF) and the treatment was ended.

## Discussion and conclusion

This case highlights the fact that EoE is now the most common cause of acute food bolus obstruction [6, 7], and that EoE is the most common cause of spontaneous perforation of the oesophagus [8]. We have not used the term Boerhaave syndrome because that was a description of a full thickness tear and pleural cavity extravasation of gastric contents [9]. The distinction is important because the majority of perforations in EoE do not have pleural extravasation of gastric content and are often smaller or partial tears and so are managed conservatively in most cases [4]. In our case the placement of a stent supported the integrity of the oesophagus while drainage and antibiotics managed the infection in the mediastinum. The literature gives guidance on the timing of stent placement, the frequency of repeat endoscopy, lavage and drainage [10, 11]. Reflecting on the timing in this case, it may be that perhaps a few days of daily endoscopy and lavage might have helped to limit the extent of the extraesophageal damage. Endoscopic VAC therapy [12] was not used in this case as the perforation was judged too large. However, it is plausible to consider that a suction sponge system in the first week, and before Stent placement might have saved this patient from subsequent thoracotomy and drainage.

The size of the tear in our case was relatively large for an EoE perforation as many examples in the literature are smaller and some resemble a cheese cloth appearance of multiple small holes, and in others a dissecting interstitial tear rather than a direct through and through perforation [4].

Another important issue highlighted by this case is the difficulty with current therapy for EoE. There are two issues – the past and the present. Until 2018 no licenced therapy in Europe (June 2019 in Sweden) was available for EoE and even today there is no licenced maintenance therapy. The patient had been treated correctly according to guidelines at the time but now we would consider that patients with daily symptoms of dysphagia should have active therapy with a proven, licenced, formulation of topical steroid such as Budesonide orodispersble table (BOT, Jorveza Dr. FalkPharma) which has been shown to induce remission of EoE in patients with moderate to severe symptoms even when other therapy with proton-pump inhibitor has failed [13]. After the stent was removed, we identified that the eosinophilic inflammation continued in this patient and therefore have now prescribed the patient a course of BOT. This has been successful in settling the mucosal eosinophilia, but probably not enough to resolve all the underlying fibrosis, which may take much longer to achieve. Acute remission will not be enough in this case and he will need maintenance therapy. Although not yet licenced it would greatly benefit this patient to take a maintenance therapy for some years in order to allow the fibrosis in the oesophagus to remodel the deeper layers back to normal. The acute resolution of eosinophils is not sufficient to return the oesophageal function to normal because of the chronic deposition of collagen and diffuse fibrosis that can occur throughout the oesophagus [14]. As was shown in the pivotal trial of BOT (Jorveza^R^; Dr. FalkPharma) the symptom improvement after a 6 week therapy is incomplete and in acute phase 12 weeks is needed to resolve the symptoms in the majority of patients. Finally, the disease of EoE is chronic and in order to maintain a normal esophageal swallowing function and thus a normal quality of life, maintenance therapy for an extended period of time is likely to be of great value. Work published in abstract form (Lucendo et al. 2019) [15] has identified the safety and efficacy of maintenance BOT therapy which may be needed in this case.

This case highlights the complication of perforation occurring during a food bolus obstruction in Eosinophilic oesophagitis. Management may be conservative but mediastinal drainage is important if significant extravasation occurs and should be instituted from the start. Definitive therapy with licenced effective topical steroid is important.

## Data Availability

Not Applicable.

## Abbrevations

EoE: Eosinophilic Oesophagitis
HPF: High Power Field
BOT: Budesonide orodispersible table
